# Spautin-1, a novel autophagy inhibitor, enhances imatinib-induced apoptosis in chronic myeloid leukemia

**DOI:** 10.3892/ijo.2014.2313

**Published:** 2014-02-27

**Authors:** Shan Shao, Su Li, Youwen Qin, Xiaorui Wang, Yining Yang, Haitao Bai, Lili Zhou, Chuxian Zhao, Chun Wang

**Affiliations:** Department of Hematology, Shanghai Jiaotong University Affiliated First People’s Hospital, Shanghai 200080, P.R. China

**Keywords:** autophagy, imatinib mesylate, spautin-1, chronic myeloid leukemia, AKT, GSK3β

## Abstract

Imatinib mesylate (IM), a targeted competitive inhibitor of the BCR-ABL tyrosine kinase, has revolutionized the clinical treatment of chronic myeloid leukemia (CML). However, resistance and intolerance are still a challenge in the treatment of CML. Autophagy has been proposed to play a role in IM resistance. To investigate the anti-leukemic activity of specific and potent autophagy inhibitor-1 (spautin-1) in CML, we detected its synergistic effect with IM in K562 and CML cells. Our results showed that spautin-1 markedly inhibited IM-induced autophagy in CML cells by downregulating Beclin-1. Spautin-1 enhanced IM-induced CML cell apoptosis by reducing the expression of the anti-apoptotic proteins Mcl-1 and Bcl-2. We further demonstrated that the proapoptotic activity of spautin-1 was associated with activation of GSK3β, an important downstream effector of PI3K/AKT. The findings indicate that the autophagy inhibitor spautin-1 enhances IM-induced apoptosis by inactivating PI3K/AKT and activating downstream GSK3β, leading to downregulation of Mcl-1 and Bcl-2, which represents a promising approach to improve the efficacy of IM in the treatment of patients with CML.

## Introduction

Chronic myeloid leukemia (CML) is a hematological malignancy characterized by the presence of the Philadelphia chromosome (1). This chimeric chromosome generates the fusion gene BCR-ABL, produces fusion proteins and leads to constitutive activation of the tyrosine kinase (TK). Deregulated TK activity results in activation of several downstream signaling pathways, including PI3K/AKT, Ras/MAPK and STAT pathways, which are implicated in mitogenic signaling and cell survival (2,3).

Treatment of CML was revolutionized by the advent of imatinib mesylate (IM), a targeted agent which inhibits the activity of BCR-ABL (4). IM has dramatically improved the prognosis of CML patients in chronic phase. However, patients in advanced stage (blast crisis) manifest drug resistance against IM, leading to relapse, due to amplification of BCR-ABL and the acquisition of BCR-ABL-independent mechanisms (5).

Autophagy is an important intracellular catabolic pathway. Autophagy helps cells to maintain cellular homeostasis. It is associated with pro-survival functions in cancer cells during cytotoxic insults such as starvation, hypoxia and chemo-therapy (6). Recent studies suggest that targeting autophagy may present a novel strategy for modulating IM resistance in CML cells including CML stem cells (7). However, the mechanism of autophagy-mediated IM-resistance remains largely unclear and strategies for targeting autophagy remain challenging.

Studies (7–9) have shown that IM induced autophagy and autophagy inhibitors boosted the therapeutic efficacy in CML cell lines such as 32D-P210 and K562. However, lack of effective small molecular inhibitors has constrained further research and clinical applications. We therefore identified a novel autophagy inhibitor named specific and potent autophagy inhibitor-1 (spautin-1), which inhibits starvation-induced autophagy in solid tumor cells (10). Whether spautin-1 affects IM-induced autophagy and cytotoxicity in CML cells is unknown. Therefore, we sought to investigate the anti-leukemic activity of spautin-1 in CML and the underlying molecular mechanisms.

We report that spautin-1 increased the cytotoxicity of IM in both K562 cell line and primary cells. Co-treatment of spautin-1 and IM enhanced cell apoptosis by inhibiting IM-induced autophagy as well as inactivating PI3K/AKT and activating downstream GSK3β, which downregulates the expression of anti-apoptotic proteins Mcl-1 and Bcl-2. Taken together, our findings suggest that when combined with spautin-1, the efficacy of IM was greatly enhanced. The combination strategy may be an interesting option to treat CML cases in the future.

## Materials and methods

### Reagents

IM was purchased from Selleckchem, USA. Spautin-1 was kindly provided by Shanghai Institute of Organic Chemistry, China Academy of Science. Both were dissolved in DMSO (Sigma-Aldrich) and stored in the dark at −20°C. LC3B (anti-rabbit, 1:2,000) was purchased from Sigma-Aldrich. Beclin-1 (anti-rabbit, 1:2,000), Bcl-2 (anti-rabbit, 1:1,000), Mcl-1 (anti-rabbit, 1:1,000) and Bim (anti-rabbit, 1:1,000) were obtained from Abcam. PARP (anti-rabbit, 1:1,000), caspase-3 (anti-rabbit, 1:1,000), AKT (anti-rabbit, 1:1,000), p-AKT^ser473^ (anti-rabbit, 1:1,000), GSK3β (anti-rabbit, 1:1,000), p-GSK3β^ser9^ (anti-rabbit, 1:1,000) and tubulin (anti-rabbit, 1:2,000) were derived from Cell Signaling Technology. ULK1 (anti-rabbit, 1:1,000), ATG5 (anti-rabbit, 1:1,000) and ATG7 (anti-rabbit, 1:1,000) were sourced from Epitomics.

### Cell culture and patients

K562 cells were generously provided by Shanghai Institute of Hematology and maintained in RPMI-1640 medium (Gibco, Life Technologies, USA) with 10% heat-inactivated fetal bovine serum (FBS) (Gibco, Life Technologies) and 1% penicillin-streptomycin (Gibco, Life Technologies) at 37°C in a humidified atmosphere containing 5% CO_2_.

The diagnosis of CML was based on clinical features, hematological characteristics and the presence of the Ph chromosome, as described previously (11). The trial was approved by Institutional Review Boards and Ethics Committees. Patients signed written informed consent, in accordance with the Declaration of Helsinki. Bone marrow mononuclear cells from CML patients were isolated by Ficoll density centrifugation. Briefly, after bone marrow aspiration, citrate-anticoagulated blood was mixed with Ficoll reagent (Cedarlane) in PBS (1:1). The leukocyte-rich plasma was subjected to density gradient centrifugation (2,000 rpm, 30 min). The cell pellet containing neutrophils was recovered and the contaminating erythrocytes were removed by hypotonic lysis with H_2_O. Cells were washed twice with PBS and kept in RPMI-1640 with 10% FBS. Cell viability, as determined by trypan blue exclusion, was consistently >95%.

### Cell proliferation assays by Cell Counting Kit-8 (CCK-8)

Cell proliferation was evaluated using CCK-8 (Beyotime). Cells (1×10^5^/ml) were seeded into 96-well plates in triplicate and then treated with 125 to 4,000 nM IM alone or in combination with spautin-1 (10 *μ*M). After 48 h of incubation, 10 *μ*l of CCK-8 reagent was added to each well. Four hours later, the absorbance was read at 450 nm using a microplate reader (Bio-Rad). The background absorbance was measured in wells containing only dye solution and culture medium. Data presented are the values subtracting the background absorbance values from the total absorbance values. The mean of the triplicates were calculated.

### Fluorescence microscopy

Apoptotic morphology was studied by staining the cells with Hoechst 33258 (KeyGen Biotech) fluorescent stain. Cells (1×10^5^/ml) were seeded into a 12-well plate with indicated concentration of IM (500 nM) for 12 h. Then spautin-1 (10 *μ*M) or DMSO was added to K562 medium for further 36 h. After incubation, cells were stained with 20 mg/ml of Hoechst 33258 for 10 min and observed under a fluorescence microscope (Olympus).

### Flow cytometry

The percentage of apoptotic cells was analyzed by flow cytometry using Annexin V-FITC Apoptosis Detection assay kit I (BD Pharmingen, Lot 25015). Cells (1×10^5^/ml) were washed with ice-cold PBS and resuspended in cold Annexin V-binding buffer containing Annexin V-FITC and propidium iodide (PI). The samples were incubated at room temperature in the dark for 10 min and the total volume was adjusted to 500 *μ*l with Annexin V-binding buffer. The number of stained cells was assessed by flow cytometer (BD FACScan). Early apoptotic cells were defined as positive for Annexin V-FITC but negative for PI staining and late apoptotic cells were positive for both Annexin V-FITC and PI staining.

Cell cycle analysis was performed by fixing cells in 70% ethanol for 12 h at 4°C, followed by incubation with 1 mg/ml RNase A for 30 min at 37°C. Subsequently, cells were stained with PI (50 *μ*g/ml) (Becton-Dickinson, San Jose, CA, USA) in phosphate-buffered saline (PBS), 0.5% Tween-20, and analyzed using a flow cytometer (BD FACScan).

### Western blot analysis

Briefly, K562 cells (1×10^5^/ml) were cultured in 6-well plates, harvested at specific intervals and lysed in lysis buffer (Beyotime). Protein concentration was measured by the BCA protein assay (Beyotime). Equal amounts of protein were resolved on 8 or 12% SDS-PAGE gel and transferred to a PVDF membrane. After blocking with phosphate-buffered saline (PBS) containing 5% non-fat milk and 0.1% Tween-20 for 2 h, membranes were incubated with primary antibodies at 4°C overnight and secondary antibodies for 2 h at room temperature. Protein-antibody complexes were detected by an enhanced chemiluminescence immunoblotting ECL (Beyotime). Immunoblots were quantified using ImageJ2x, and the levels of protein were normalized to tubulin levels.

### Statistical analysis

All assays were performed in triplicate and data expressed as mean values ± SD. Statistical significance of differences between groups was determined using Student’s t-test. Probability value ≤0.05 was considered significant and marked by asterisks in the figures. All statistical analysis was conducted using SPSS 13.0.

## Results

### Spautin-1 inhibits IM-induced autophagy in K562 cells

**Figure 1.**
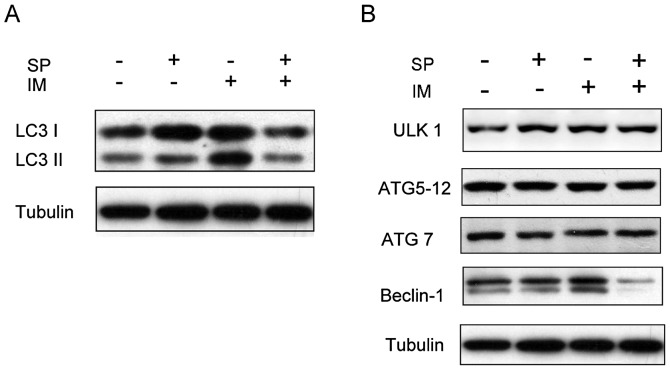

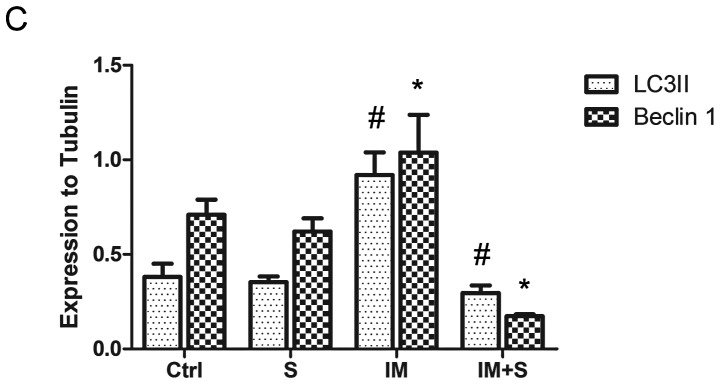
Spautin-1 inhibits IM-induced autophagy in K562 cells. After treatment with or without IM (250 nM) for 12 h, spautin-1 (10 *μ*M) or DMSO (0.1%) was added to K562 medium for further 36 h. (A) Autophagy was denoted by the switch of LC3I to LC3II, which was detected by western blotting (WB). (B) Other autophagy factors such as Beclin-1, ULK1, ATG 5 and ATG 7 were also detected by WB. (C) The bar chart demonstrates the ratio of LC3II and Beclin-1 proteins to tubulin by densitometry. Data are expressed as mean ± SD (^#,*^P≤0.05 between groups).

Previous studies reported autophagy underlying IM resistance in CML cells (8,12,13). To investigate whether spautin-1 affected IM-induced autophagy, we determined microtubule-associated protein light chain 3 (LC3) conversions (LC3-I to LC3-II) by immunoblot analysis. LC3 is now widely used to monitor autophagy. The amount of LC3-II correlates with the number of autophagosomes. Autophagy was activated in K562 cells after treatment with 0.5 *μ*M IM for 48 h, which was consistent with previous reports (9). Spautin-1 showed strong inhibition of IM-induced autophagy in K562 cells (Fig. 1A and C). We also observed the expression of a few autophagy-related factors, such as Beclin-1, ULK1, ATG5-12 complex and ATG7 in K562 cells. The results showed that spautin-1 markedly attenuated the protein levels of Beclin-1 induced by IM, but had no significant effect on other autophagy factors (Fig. 1B and C). These findings suggested that spautin-1 inhibited IM-induced autophagy in a Beclin-1-dependent manner.

### Spautin-1 enhances IM-induced cytotoxicity in K562 cells

**Figure 2.**
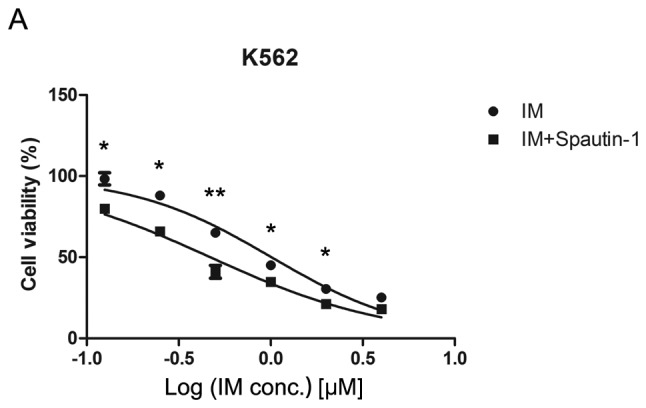

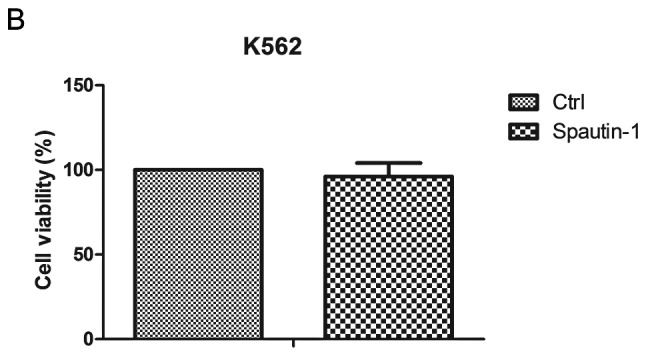

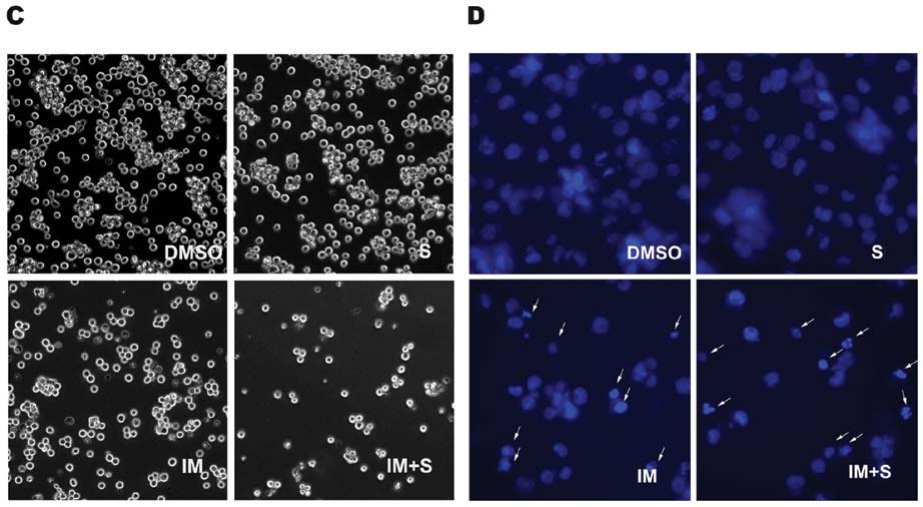

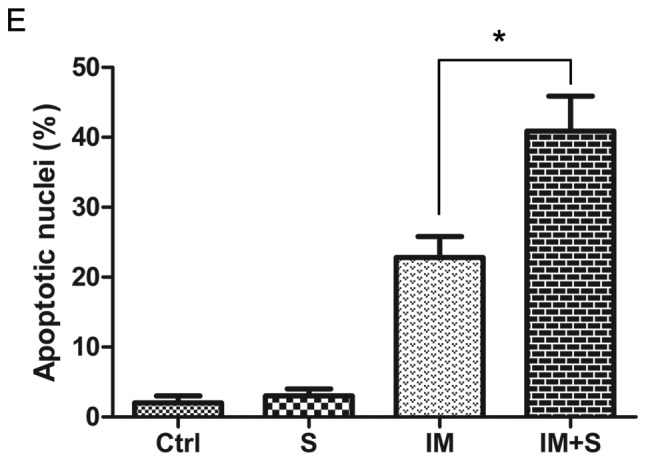
Spautin-1 enhances IM-induced cytotoxicity in K562 cells. (A) Cells were treated with varying concentrations of IM for 48 h in the presence or absence of spautin-1 (10 *μ*M). Cell viability was determined by CCK-8 assay. Data are expressed as the mean ± SD, and analyzed by Student’s t-test (^*^P<0.05 and ^**^P<0.01). (B) Cell viability of spautin-1 alone (10 *μ*M) group compared with control group after 48-h incubation. Cell viability was determined by CCK-8 assay. After treatment with 500 nM IM or DMSO for 12 h, spautin-1 (10 *μ*M) or DMSO was added to K562 medium for further 36 h. (C) Changes in cellular morphology were examined by a light microscope (×200). (D) Cells were stained with Hoechst 33258 fluorescent stain. Changes were observed under a fluorescence microscope (×400). Arrows point to the apoptotic nuclei. (E) Apoptotic nuclei were quantified by counting 10^2^ cells on three separate fields for each condition. Results from three independent experiments are shown. Data are expressed as mean ± SD (^*^P≤0.05).

Several studies demonstrated that IM-induced autophagy was a protective response preventing CML cells from therapy-induced cell death (7,14). The observed effect of spautin-1 in autophagy suggested that it rendered CML cells increasingly susceptible to IM. To verify this idea, we compared the growth inhibitory effect of IM on K562 cells before and after inhibition of autophagy by spautin-1. K562 cells were treated with varying concentrations of IM in the presence or absence of 10 *μ*M spautin-1 for 48 h. It was followed by CCK8 assay and morphological examination of the treated cells. As shown in Fig. 2A, IM inhibited the growth of K562 cells with 50% inhibition (IC_50_) of 1.03 *μ*M at 48 h. In contrast, co-treatment with spautin-1 increased IM-induced inhibition of cell viability with IC_50_ of 0.45 *μ*M. Spautin-1 alone showed no significant impact on cell viability after 48 h of incubation (Fig. 2B). Microscopic evidence indicated that IM combined with spautin-1 significantly boosted IM-induced cell death (Fig. 2C). These results were confirmed by fluorescent microscopy of Hoechst 33258 staining (Fig. 2D and E). The data suggested that autophagy inhibitor spautin-1 enhanced IM-induced cytotoxicity in K562 cells.

### Spautin-1 enhances IM-induced apoptosis in K562 cells

**Figure 3.**
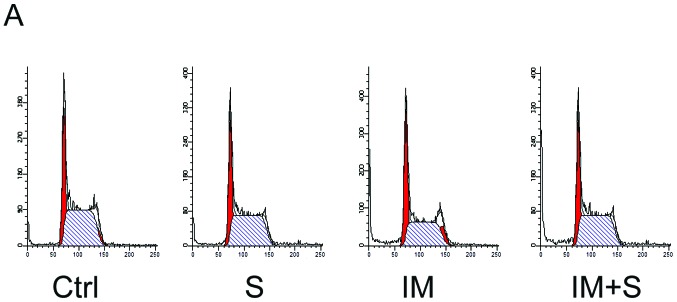

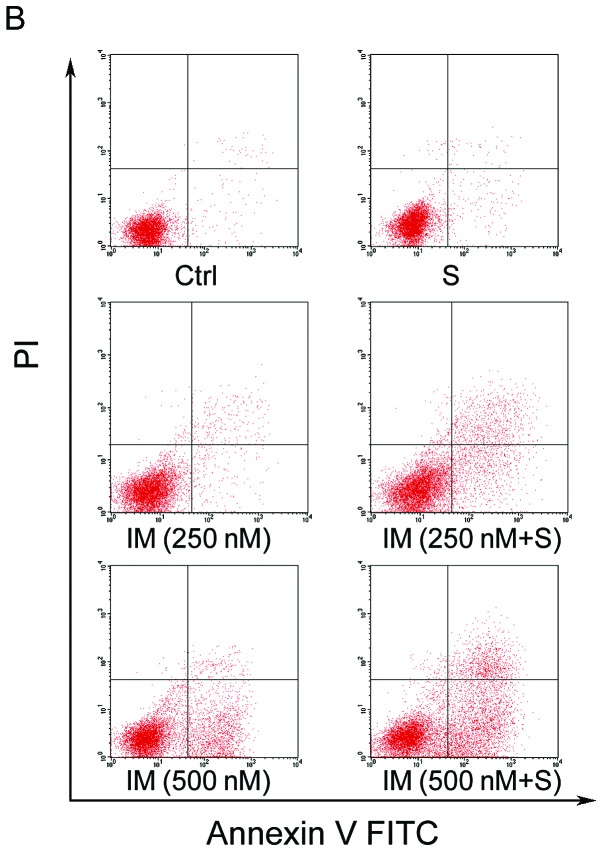

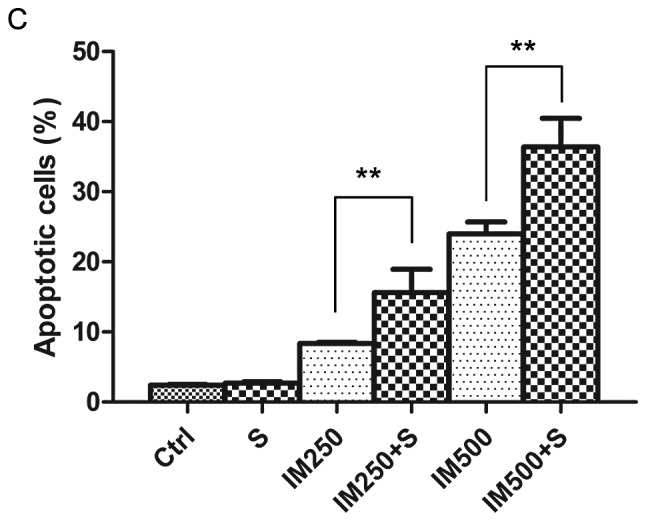

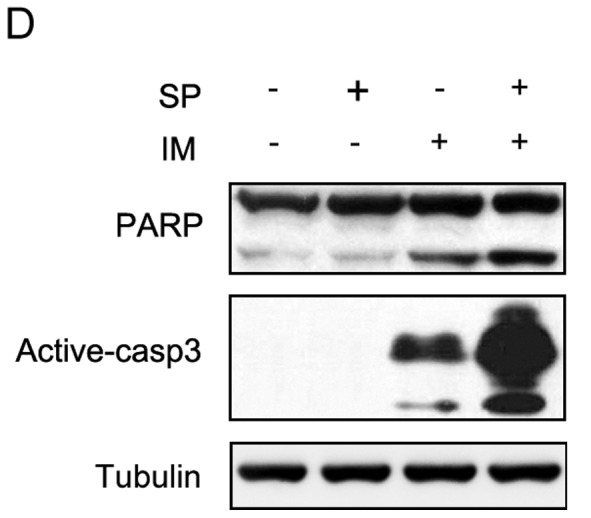

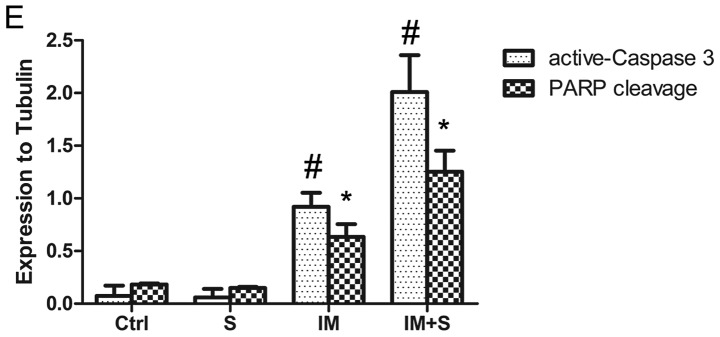

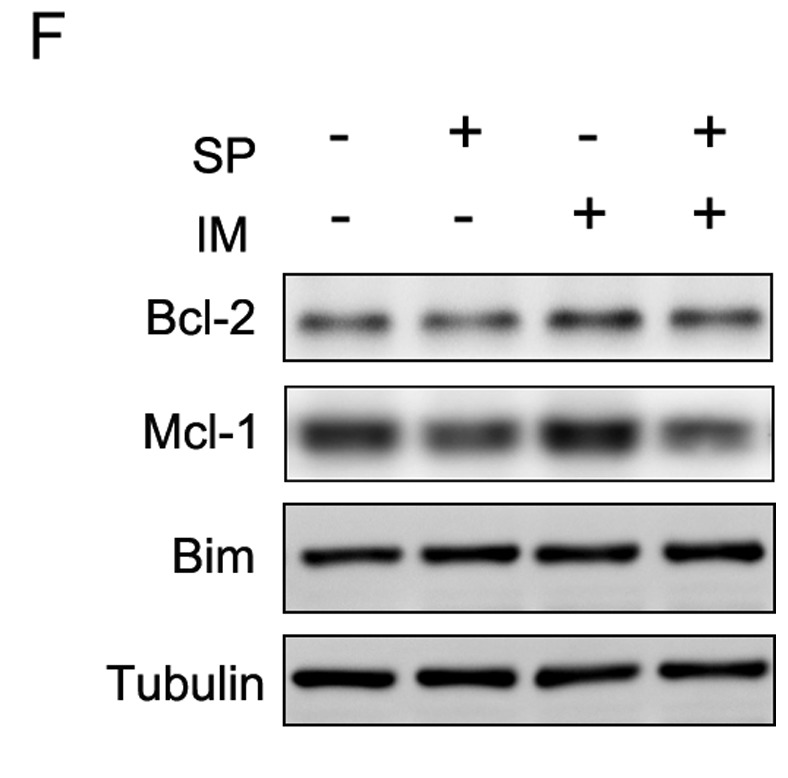
Spautin-1 promotes IM-induced apoptosis in K562 cells. (A) K562 cells were treated with IM (500 nM) or DMSO (0.1%) for 12 h and then incubated with or without spautin-1 (10 *μ*M) for further 36 h. Cells were analyzed by flow cytometry for cell cycle measurement. Results from the single experiment represent all the three experiments. K562 cells were treated with IM (250 or 500 nM) or DMSO (0.1%) for 12 h and then incubated with or without spautin-1 (10 *μ*M) for further 36 h. (B and C) The apoptotic cells were quantified by Annexin V-PI staining using flow cytometry. Representative FACS data and a summary of three experiments are depicted. Data are mean ± SD (^**^P≤0.01). (D) Western blot analysis of caspase-3 and PARP cleavage. (E) The bar chart demonstrates the ratio of caspase-3 and PARP cleavage proteins to tubulin, by densitometry. Data are expressed as mean ± SD (^*,#^P≤0.05). (F) Western blot analysis of Mcl-1, Bim and Bcl-2. Tubulin served as a loading control. Representative blots of three independent experiments are shown.

We further investigated whether spautin-1 affected cell cycle or cell death in IM-treated K562 cells. Cell cycle and apoptosis were analyzed by flow cytometry after staining with PI and PI/Annexin V, respectively. No sign of obvious cell cycle arrest was found in any group (Fig. 3A). However, after treated with 250 or 500 nM IM for 48 h, with or without spautin-1, co-treatment with spautin-1 enhanced IM-induced apoptosis from 8.4 and 23.9% to 15.5 and 36.4%, respectively (Fig. 3B and C). Additionally, spautin-1 alone showed no significant impact on apoptosis. We also found that spautin-1 increased IM-induced caspase-3 cleavage, which was an indicator of cell apoptosis. Consistently, the level of cleaved product of caspase substrate poly(ADP-ribose) polymerase (PARP) correlated with the activation of caspase (Fig. 3D and E). To investigate the mechanism of spautin-1 in promoting IM-induced apoptosis of K562 cells, we focused on Bcl-2 family proteins, which played a key role in cell apoptosis. Compared with IM alone, co-treatment of spautin-1 and IM remarkably downregulated anti-apoptotic proteins including Bcl-2 and myeloid cell leukemia-1 (Mcl-1) (Fig. 3F). These data collectively illustrated that spautin-1 promoted IM-induced CML cell apoptosis by reducing the expression of anti-apoptotic proteins Bcl-2 and Mcl-1.

### AKT/GSK3β is involved in spautin-1 pro-apoptotic activity in CML cells

**Figure 4.**
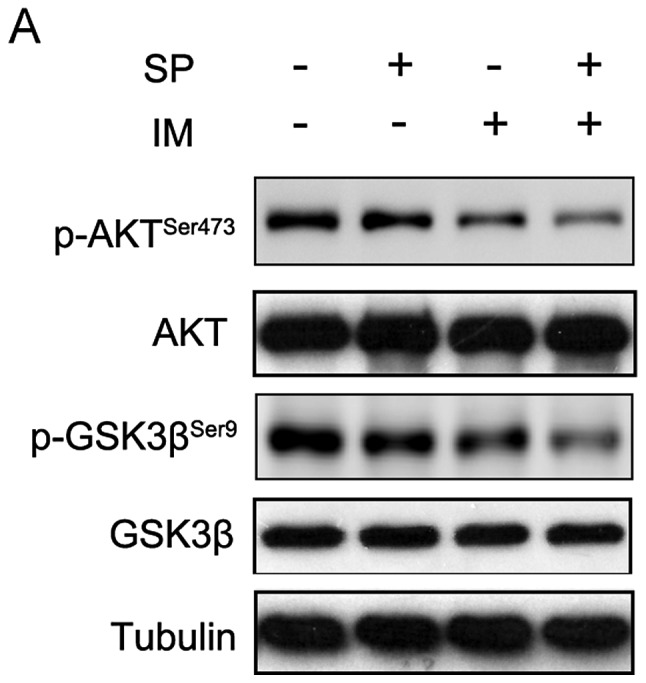

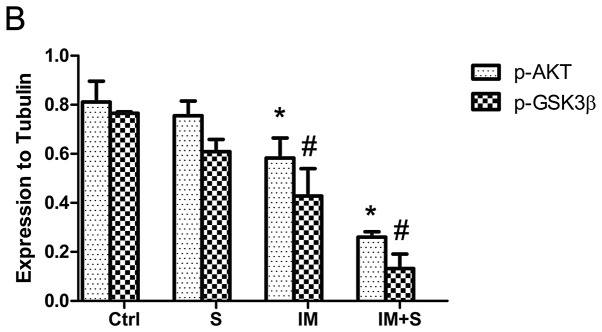
AKT/GSK3β is involved in spautin-1 pro-apoptotic activity in CML cells. K562 cells were treated with IM (250 nM) or DMSO (0.1%) for 12 h and then incubated with or without spautin-1 (10 *μ*M) for 36 h. (A) Total AKT, p-AKT^ser473^, total GSK3β and p-GSK3β^ser9^ levels were evaluated by WB. Tubulin served as a loading control. Representative blots of three independent experiments are shown. (B) The bar chart demonstrates the ratio of AKT^ser473^ and GSK3β^ser9^ proteins to tubulin by densitometry. Data are expressed as mean ± SD (^*,#^P≤0.05).

We determined the mechanism underlying regulation of IM-induced CML cell apoptosis by spautin-1. Accumulating evidence supports that PI3K/AKT/mTOR signaling pathway was involved in autophagy and apoptosis (15–17). We investigated whether this pathway functioned in inhibiting autophagy and promoting the apoptosis by spautin-1 in IM-treated K562 cells. As shown in Fig. 4A, the combination of IM and spautin-1 sharply suppressed AKT^ser473^ phosphorylation compared with IM alone. The total AKT level was unchanged. AKT comprises many kinase substrates with diverse functionalities. We detected some identified ATK substrates, such as p27, FOXO and GSK3β. Interestingly, GSK3β^ser9^ phosphorylation was also markedly reduced in IM/spautin-1 co-treated K562 cells compared with cells treated with IM alone (Fig. 4). In each group, total GSK3β was unaffected. Phosphorylation at Ser9 is a major mechanism for inhibiting GSK3β enzymatic activity (18). These results indicated that the suppression of AKT and activating downstream GSK3β were involved in autophagy inhibition and promotion of apoptosis in spautin-1/IM co-treated CML cells.

### Spautin-1 potentiates the efficacy of IM in primary CML cells

**Figure 5.**
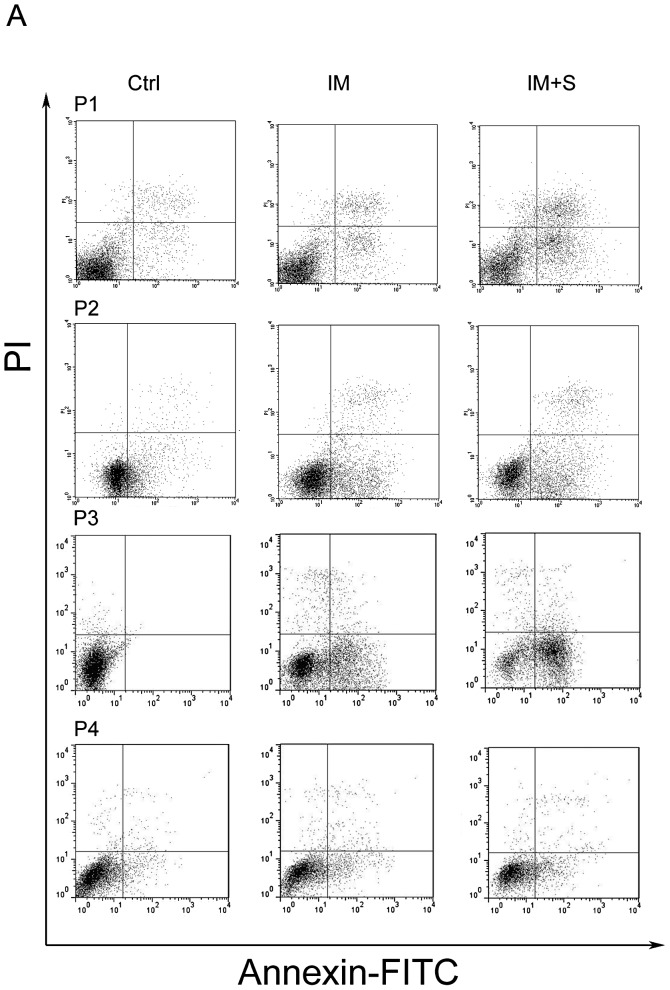

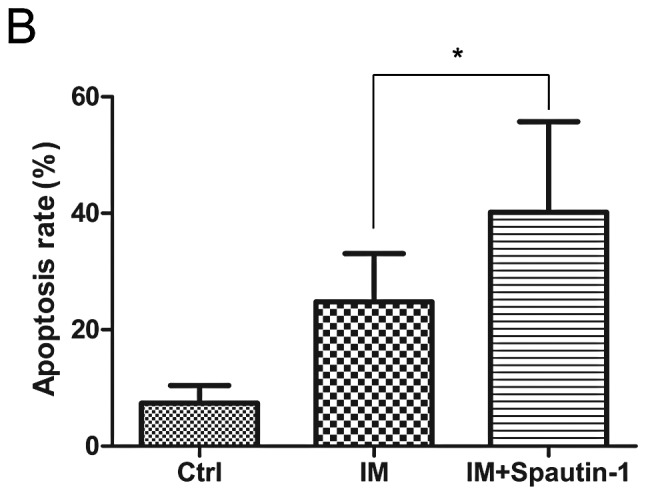
Spautin-1 potentiates the efficacy of IM in primary CML cells. Flow cytometry results of primary CML cells treated with IM with or without spautin-1. Primary cells were treated with IM (2 *μ*M) or DMSO (0.1%) for 12 h and then incubated with or without spautin-1 (10 *μ*M) for 36 h. (A) The apoptotic cells were quantified by Annexin V-PI staining using flow cytometry. (B) The bar chart demonstrates the ratio of apoptotic cells among the groups. Data are expressed as mean ± SD (^*^P<0.05 between groups).

The aforementioned results indicated that spautin-1 enhanced the cytotoxic sensitivity of IM in CML cell line K562. We therefore, wondered whether spautin-1 affected primary CML cells similarly. Isolated primary cells from four CML patients were used to assess the pro-apoptotic activity of spautin-1. Cell apoptosis was analyzed by flow cytometry with PI/Annexin V staining. Consistently with findings in K562 cells, combination of IM and spautin-1 significantly promoted cell death in primary cells. The mean apoptotic rate was 40.2±11.4% and 24.8±6.9% in the combination group and IM alone group, respectively (P<0.05) (Fig. 5). The above data indicated that spautin-1 enhanced IM-induced cell death both in K562 and primary CML cells.

## Discussion

IM is associated with a substantial therapeutic effect in CML and has been endorsed as the first line therapy in CML by international guidelines (19). However, CML cells may respond to IM in a variety of ways ranging from initiation of cell death to the activation of survival pathways such as autophagy (7,20,21). In our study, the novel autophagy inhibitor, spautin-1, exhibited significant efficacy in enhancing IM-induced cytotoxicity in both K562 and CML cells.

Several studies have shown that the blockage of autophagy by pharmacologic inhibitors or genetic knockdown of critical autophagy-related genes enhanced the cytotoxicity of IM on CML cells (12). Considering the crucial role played by autophagy in the treatment of CML and the shortage of effective autophagy inhibitors, we studied the efficacy of spautin-1, which has been identified to inhibit starvation-induced autophagy. We observed that spautin-1 markedly inhibited IM-induced autophagy by downregulating the autophagy protein Beclin-1. The Beclin1/VPS34 complex plays a crucial role in autophagosome formation. In fact, Beclin-1 has been implicated in the progression of solid tumors and leukemia (22). Earlier studies reported that inhibition of autophagy by knockdown of Beclin-1 sensitized CML cells to chemo-therapy drugs (7,23). Spautin-1 specifically targeted Beclin-1 and inhibited IM-induced autophagy, which may contribute to enhanced effect of IM in CML. We found that spautin-1 enhanced IM-induced cytotoxicity in CML cell line K562, decreasing the IC_50_ from 1 to 0.5 *μ*M. This result was also confirmed in primary cells from CML patients. Therefore, spautin-1 sensitized the CML cells to IM cytotoxicity, which correlated with autophagy inhibition.

We investigated the cell cycle and apoptosis in K562 cells and found that spautin-1 significantly promoted the IM-induced apoptosis of K562 cells in a caspase-dependent manner but did not change cell cycle distribution compared with IM alone. Previous studies have shown that IM induced CML cell apoptosis mainly by activating Bcl-2 family proapoptotic proteins: Bim and Bad (24). However, Bim was not affected either by IM alone or combined with spautin-1 in our experiments. This phenomenon may be attributed to a low concentration of IM (0.5 *μ*M). Interestingly, the combination of IM and spautin-1 significantly downregulated the anti-apoptotic protein Bcl-2 and Mcl-1 compared with IM alone. This indicated that spautin-1 promoted IM-induced CML cell apoptosis by downregulating anti-apoptotic protein Bcl-2 and Mcl-1.

To further elucidate the mechanism of the synergistic effect of spautin-1 and IM, we focused on the PI3K/AKT signaling pathway, which regulated apoptosis and autophagy. Our results showed that IM partially reduced the phosphorylation level of AKT^Ser437^, leading to decreased activity of AKT. Interestingly, co-treatment of IM and spautin-1 further inhibited the phosphorylation of AKT compared with IM alone. We found spautin-1 alone inhibited the phosphorylation of AKT. The PI3K/AKT pathway was frequently activated in leukemia, promoting survival and preventing apoptosis in CML cells, especially in IM-resistant cells (25). It has been reported that treatment with PI3K/ATK pathway inhibitor effectively inhibited the resistance to IM of CML cells (17). Our results suggested that spautin-1 might promote IM-induced apoptosis in CML cells by inactivating AKT.

Among numerous AKT downstream substrates, we focused on mTOR, FOXO1, Bad, p27 and GSK3β, which correlated with cell apoptosis and proliferation. It has been established that Ser9 dephosphorylation of GSK-3β results in its activation and is associated with cell apoptosis (26). Our results showed that GSK3β^Ser9^ phosphorylation level was significantly downregulated by co-treatment of spautin-1 and IM, indicating that GSK3β was actually activated. The active GSK3β triggers phosphorylation-mediated proteasomal degradation of the anti-apoptotic protein Mcl-1 (27–29). It has been reported that downregulation of Mcl-1 through GSK-3β activation contributes to chemically-induced apoptosis in acute myeloid leukemia cells (30). We also found that Mcl-1 protein level in K562 cells was markedly reduced when co-treated with spautin-1 and IM, but not when treated with IM alone. Therefore, it is suggested that spautin-1 activated GSK3β^Ser9^ by inactivating AKT, which ultimately resulted in downregulation of the anti-apoptotic protein Mcl-1.

In conclusion, spautin-1 enhanced IM-induced cytotoxicity in K562 cell line. Spautin-1 inhibited IM-induced autophagy and restrained the pathway, which led to CML cell survival. On the other hand, spautin-1 promoted cell apoptosis by activating GSK3β through PI3K/AKT, which reduced the anti-apoptotic protein Mcl-1. Spautin-1 was also effective in primary CML cells.

Altogether, our study indicates that spautin-1 is a promising new approach for CML treatment in combination with IM. Additional studies of the signaling pathways related to autophagy and cell death, especially the AKT/GSK3β should provide effective therapeutic strategies against CML.
